# Prenatal Exposure to Gutkha, a Globally Relevant Smokeless Tobacco Product, Induces Hepatic Changes in Adult Mice

**DOI:** 10.3390/ijerph17217895

**Published:** 2020-10-28

**Authors:** Shannon Doherty Lyons, Jason L. Blum, Carol Hoffman-Budde, Pamela B. Tijerina, M. Isabel Fiel, Daniel J. Conklin, Francesca Gany, Joseph A. Odin, Judith T. Zelikoff

**Affiliations:** 1 Department of Environmental Medicine, New York University School of Medicine, New York, NY 10010, USA; shannon.doherty@nyulangone.org (S.D.L.); jasblum@gmail.com (J.L.B.); roscoe10924@yahoo.com (C.H.-B.); pbt217@gmail.com (P.B.T.); 2 Product Safety Labs, Dayton, NJ 08810, USA; 3 Department of Pathology, Molecular and Cell-Based Medicine, Icahn School of Medicine at Mount Sinai, New York, NY 10029, USA; mariaisabel.fiel@mountsinai.org; 4 American Heart Association-Tobacco Regulation and Addiction Center, University of Louisville, Kentucky, KY 40202, USA; dj.conklin@louisville.edu; 5 Department of Psychiatry and Behavioral Sciences, Memorial Sloan-Kettering Cancer Center, New York, NY 10065, USA; ganyf@mskcc.org; 6 Department of Medicine, Icahn School of Medicine at Mount Sinai, New York, NY 10029, USA

**Keywords:** smokeless tobacco, developmental origins of health and disease, gutkha, hepatic, liver disease

## Abstract

Maternal exposures during pregnancy affect the onset and progression of adult diseases in the offspring. A prior mouse study indicated that maternal tobacco smoke exposure affects hepatic fibrosis in adult offspring. Gutkha, a broadly used smokeless tobacco (ST) product, is widely used by pregnant woman in many countries. The objective of this murine study was to evaluate whether oral maternal exposure to gutkha during pregnancy alters non-alcoholic fatty liver disease (NAFLD) in adult offspring: risk factors for the progression of NAFLD to cirrhosis in adults remain elusive. Buccal cavity ‘painting’ of pregnant mice with gutkha began on gestational days (GD) 2–4 and continued until parturition. Beginning at 12 weeks of age, a subset of offspring were transitioned to a high-fat diet (HFD). Results demonstrated that prenatal exposure to gutkha followed by an HFD in adulthood significantly increased the histologic evidence of fatty liver disease only in adult male offspring. Changes in hepatic fibrosis-related cytokines (interleukin (IL)-1b and IL-6) and in hepatic collagen mRNA expression were observed when comparing adult male offspring exposed to gutkha in utero to those not exposed. These findings indicate that maternal use of gutkha during pregnancy affects NAFLD in adult offspring in a sex-dependent manner.

## 1. Introduction

The estimated global prevalence of non-alcoholic liver disease (NAFLD) is currently 25% in the adult population [1] which parallels the increase in obesity and the spread of high-fat western diets. The spectrum of NAFLD ranges from simple steatosis (i.e., fatty liver), through steatohepatitis (SH; fatty liver with inflammation), to cirrhosis (i.e., liver inflammation with fibrosis). Approximately 7–30% of those with NAFLD have non-alcoholic steatohepatitis (NASH) [1]. It remains unclear why only a proportion of those with NAFLD have more advanced NASH. Cytokine activation of collagen-producing hepatic stellate cells leads to increased hepatic inflammation and fibrosis characteristic of NASH [2]. Stopping hepatic fibrosis is critical to preventing cirrhosis. Both environmental and genetic factors likely affect this activation process. 

Given that early-life exposures to certain chemicals can produce delayed/persistent adverse health outcomes (e.g., obesity), prenatal exposures may have a delayed effect on NAFLD in adults. Indeed, studies performed previously in this laboratory indicated that tobacco smoke exposure in mice during pregnancy predisposed the offspring to liver fibrosis in adulthood ([3]). Because placental blood first passes through the fetal liver, the liver may be particularly susceptible to later-life effects of maternal toxicant exposure during pregnancy.

While tobacco smoking during pregnancy is strongly discouraged, the prevalence of tobacco use in South Asians living in the US is estimated to exceed 25%, specifically when referring to culturally specific smokeless tobacco (ST) products, such as gutkha, paan, or bidis ([4]). ST use by women has traditionally been culturally acceptable and even encouraged during pregnancy as a remedy for morning sickness [5,6]. Unfortunately, the use of ST during pregnancy has been closely linked to an increased risk for stillbirth and preterm birth and to a 2–3 times higher risk for low birth weight [7,8,9,10]). While the adverse effects of traditional cigarette smoking on the liver is well documented [11,12,13,14], the effects of ST, such as gutkha, on the hepatic system are poorly defined, and even less is known concerning the hepatic effects on offspring exposed prenatally, despite the approximately 250 million adults that consume gutkha in the SE Asian region alone. Gutkha is a mixture of betel leaf, areca nut, slaked lime, catechu, spices, sweet or savory flavorings (aka betel quid) [15], and dried tobacco. All of the betel quid, paan, or gutkha mixtures are classified as Group 1 carcinogens by IARC [16] due to the inclusion of areca nut and betel leaf/betel quid. In addition to the carcinogenicity of these products, gutkha contains a high level of nicotine, specifically, the Rasikal Manikchand Dhariwal or ‘RMD’ brand of gutkha used for these studies (11–12 mg nicotine/packet) and marketed on-line (http://www.ntpd.org.uk/RMD_Gutkha [17]). 

Studies previously conducted in this laboratory [18] have shown that gutkha exposure in mice, even in the short term, causes changes in body weight gain and in heart and liver weights, as well as an increase in hepatic CYP2A5 gene expression [18], which is upregulated in various liver diseases, including NAFLD [19,20]. Since ST is usually chewed or sucked slowly and kept in the oral cavity for long periods of time, it becomes more aqueous, and oral absorption, as well as ingestion and absorption into the systemic circulation, are strong possibilities [21]. Consequently, many ST ingredients, including nicotine, enter the maternal circulation and cross the placenta to affect the fetus. Thus, the current murine study investigated the later-life hepatic effects of maternal exposure to gutkha during pregnancy on adult male and female offspring. Given the increasing rise worldwide of the availability and use of culturally specific ST products like gutkha and their known use during pregnancy, it is crucial not only to identify potential maternal and fetal health effects but also to investigate later health-related effects in adult offspring of both sexes.

## 2. Materials and Methods 

### 2.1. Animals

Male (for breeding purposes only) and female B_6_C_3_F_1_ mice (Jackson laboratories, Bar Harbor, ME, USA; 8–9-week-old) were maintained on a 12 h light/dark cycle and housed 1–2 per cage. All animal procedures were conducted under an animal protocol approved by the NYULMC Institutional Animal Care and Use Committee (IACUC)(#141221-01).

### 2.2. Breeding and Gutkha Preparation/Exposure 

**Figure 1:**
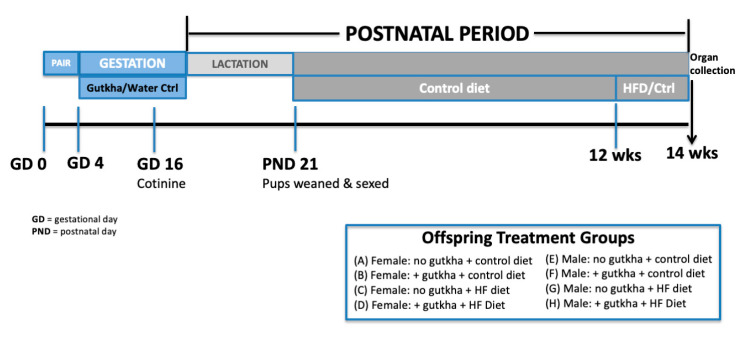
Experimental design and exposure timeline. GD = gestational day, PND = post-natal day.

As outlined in Figure 1, female B_6_C_3_F_1_ mice were paired with males for 2–4 nights prior to oral gutkha exposure. Gutkha was prepared as previously published by [18].

In brief, commercially available gutkha (RMD, India: http://www.manikchandgroup.com/rmd/gutkha.html; purchased via the internet) was steeped in a closed flask with distilled water and placed in a 37 °C shaker bath (125 rpm for approximately 18 hrs) to release the water-soluble components. Afterwards, the material was filtered (Corning^TM^ disposable vacuum filter) to remove insoluble particles, and the filtrate was lyophilized, aliquoted, and stored at −80 °C to assure stability until use. The sample, allowed to reach room temperature, was prepared daily by dissolving the lyophylate in double-distilled (dd) water at a concentration of 0.425 mg/µl. Fifty microliters of the resulting gutkha solution or double-distilled water vehicle (control) was administered by ‘painting’ the upper and lower palate and tongue of each mouse using a natural bristle brush. Gutkha administration began on gestational day (GD) 2–4 and continued until parturition (at or about GD20; n = 5–14 litters per treatment group). Dams were weighed daily throughout pregnancy; offspring were weighed from birth through post-natal day (PND) 12. Gestational parameters such as incidence of pregnancy (defined as the number of litters at birth/number of dams), litter size, and sex ratios were measured post-partum.

### 2.3. Cotinine Measurements

Serum was collected from the dams on GD 16, and cotinine levels were measured immediately using a commercially available ELISA kit (Orasure, PA, USA). Cotinine levels were also measured in the amniotic fluid and in the fetal liver on GD 16 using the same assay system and a separate cohort of pregnant mice. The latter cohort included 5 dams/treatment group; cotinine levels in fetal livers were measured by pooling 3 fetal livers/dam/treatment group. 

### 2.4. High-Fat Diet

All prenatally exposed gutkha offspring of both sexes and their sex-matched dd water control counterparts were weaned onto a normal (regular) chow diet (13% of calories from fat; LabDiet 5001) for the first 12 weeks after birth. Half of the offspring from each prenatal treatment group were switched to a high-fat diet (HFD) (42% calories from fat (Harlan TD.88137)) for 2 weeks beginning at 12 weeks of age; mice not subsequently fed a HFD were maintained on normal chow for the same time interval. At the end of the 2-week HFD exposure, 14-week-old male and female offspring were fasted overnight (approximately 18 hrs) and euthanized using an overdose of SleepAway (120 mg/kg BW). Blood and selected organs (i.e., liver, thymus, heart) were then collected from adult male and female offspring from each treatment group (8 groups total; n = 5–9 mice from each sex/treatment group).

### 2.5. Histopathology 

The liver, thymus, and heart were weighed, fixed in 10% formalin overnight, and then embedded in paraffin and sectioned. Five-mm liver sections were stained either with hematoxylin and eosin (H&E) or with 0.1% Sirius red F3B (Sigma, St. Louis, MO, USA) in saturated picric acid (Sigma) [22]. H&E-stained sections were scored for steatohepatitis grade using the NASH activity score (NAS), which is defined as the sum of the scores for steatosis (0–3), lobular inflammation (0–3), and ballooning (0–2) [23]. Thus, the results were a continuum ranging from 0 to 8. NAS scores of 0–2 are considered not diagnostic of NASH, while scores of 3–4 are considered possible NASH, and scores of 5–8 are considered diagnostic of NASH. Fibrosis, believed to be a consequence of steatohepatitis, is measured separately from the NAS on a scale ranging from stage 0 to stage 4, with stage 4 indicative of cirrhosis. All liver sections were analyzed and scored in a blinded manner by a trained liver pathologist (Icahn School of Medicine at Mount Sinai Department of Pathology). 

### 2.6. Semi-Quantitative RT-PCR

Real-time polymerase chain reaction (semi-quantitative RT-PCR) was performed to determine changes in hepatic mRNA gene expression in adult offspring associated with gestational gutkha exposure, combined (or not) with a subsequent HF-diet. Gene expression of collagen 1A1 (Col1A1), tumor necrosis factor (TNF)-α, interleukin (IL)-1b, and IL-6, with 18S rRNA as the house-keeping gene (see Supplemental Table S1 for a list of primers), were analyzed as performed previously in this laboratory [24]. RT-PCR data are presented as normalized (delta) threshold cycle (dCt) values and were analyzed as described in the Statistics section.

### 2.7. Plasma Lipid Analyses

Blood plasma levels of high- and low-density lipoproteins (HDL and LDL, respectively), triglycerides (TRIG), and liver injury markers (alanine and aspartate aminotransferases [ALT and AST, respectively]) were measured using a Cobas Mira Plus automated clinical chemistry analyzer (Roche Diagnostic Systems, Inc., Branchburg, NJ, USA), as previously published [25]. 

### 2.8. Statistics

Biological parameters were analyzed by one-way analysis of variance (ANOVA) followed by post-hoc testing (when appropriate), using the SPSS (IBM, Armonk, NY, USA, 18.0 statistics) package. Real-time PCR values were compared for each treatment group using the dCt values for each target gene. This particular method was selected to enable comparisons between appropriate control and treatment groups (i.e., within-diet comparisons between gutkha-exposed and control mice or within-maternal exposure comparisons between diets). The values were calculated by subtracting the Ct value for the 18S rRNA from the Ct values for each gene of interest. Real-time PCR expression data, presented as dCt values, were used for comparisons between selected treatment group pairs using one-way ANOVA, whereas lower dCt values represent higher levels of mRNA expression. In the text, fold changes were computed using the standard formula, 2^−ddCt^. Significance between the groups was accepted at *p* < 0.05. 

## 3. Results

### 3.1. Cotinine Measurements 

Maternal serum cotinine levels were measured on GD 16 and used as a biomarker of gutkha exposure. As expected, cotinine levels were below detection levels in sham-exposed control dams. Cotinine levels in the amniotic fluid of gutkha-exposed dams were similar to those measured in the serum of similarly exposed dams (i.e., 38 and 39 ng/mL, respectively). In contrast, cotinine concentrations in the fetal liver were approximately 100-fold lower (0.31 ng/mg) than those measured in the amniotic fluid or maternal serum. 

### 3.2. Gestational Parameters 

Maternal oral exposure to gutkha throughout gestation had no significant effect on the incidence of full-term pregnancies compared with that of control dams (73% vs. 85%, respectively; *p* > 0.05). Likewise, gutkha exposure during gestation had no significant effect on the average number of viable pups per litter (8–9 pups). However, significantly more male than female pups (*p* < 0.01) were born to gutkha-exposed pregnant mice as compared with the number of males born to control dams (male/female ratio in gutkha-exposed litters = 1.32 vs. 0.74 in control litters). Additionally, exposure to gutkha had no significant effect on dam weight gain throughout gestation, compared with GD-matched controls; both control dams and those exposed to gutkha during pregnancy nearly doubled their body weight over the same time period (i.e., all mice gained ~90% of their pre-pregnancy body weight by parturition). 

### 3.3. Offspring Body and Organ Weights

**Figure 2:**
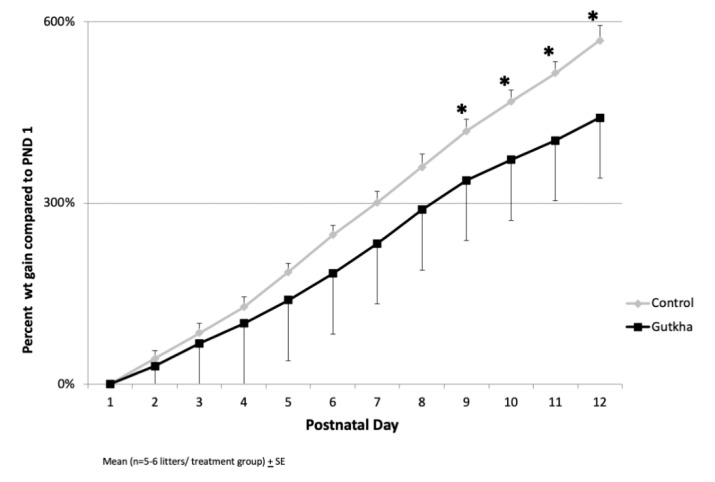
Prenatal exposure to gutkha significantly reduced offspring weight gain (* *p* < 0.05) over time up to PND 12.

While exposure to gutkha during pregnancy did not affect offspring birth weight, prenatal gutkha exposure led to a slower rate of weight gain (n = 5–6 litters/treatment group) compared with age-matched control offspring (Figure 2). Due to the gutkha-associated slower rate of weight gain, the differences in body weight between groups reached statistical significance (*p* < 0.05) by PND 9. 

There were no significant differences in the average body weight of male and female offspring in either exposure group at 12 weeks of age prior to the start of the HFD (male offspring body weight: 39.91 g and 39.19 g for control and gutkha groups, respectively, *p* > 0.05; female offspring body weight: 28.23 g and 26.17 g for control and gutkha groups, respectively, *p* > 0.05). However, at the time of sacrifice (14 weeks of age), female offspring prenatally exposed to gutkha alone (without HFD) had a significantly lower (*p* < 0.002) absolute body weight compared with their sex-matched, non-gutkha-exposed counterparts (23.94 g vs. 31.44 g), which was an unexpected result based on the lack of change in body weight at 12 weeks. Both male and female offspring fed HFD (42% of calories from fat) for 2 weeks (from 12 to 14 weeks of age) gained more weight than their gutkha-/non-gutkha matched control group maintained on a regular normal chow diet (Table 1). Weight gain was significantly greater (*p* < 0.03) in the non-gutkha exposed (control) female offspring fed the HFD (group C) compared with female offspring counterparts fed the regular diet (group A). The weight gain of male mice not exposed to gutkha and fed the HFD (group G) was significantly less in comparison with that of the other groups fed the HFD (groups C and H, *p* < 0.04 and *p* < 0.03, respectively); male offspring fed the HFD following prenatal gutkha exposure (Group H) had a significantly greater weight compared with those exposed to gutkha alone (Group F, *p* < 0.03).

### 3.4. Adult Offspring Liver Histopathology

**Figure 3:**
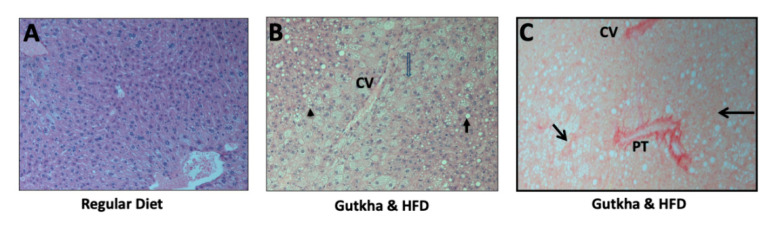
**Stained Liver Sections**. (**A**) H&E staining of mice fed normal diet showed little or no steatosis or fibrosis. (**B**) H&E staining of some mice, particularly males exposed to gutkha in utero and then fed a HFD, showed evidence of microsteatosis (arrowhead), macrosteatosis (black arrow) and fibrosis (open arrow). (**C**) Sirius red staining of collagen helps to highlight areas with a “chicken wire” pattern of fibrosis (black arrows) typical of non-alcoholic steatohepatitis. Representative images (100×) from male offspring are shown. CV-central vein. PT-portal tract.

H&E staining of liver sections was performed in order to evaluate each treatment group for NAFLD using the NAS [26]. Representative images showed the relative paucity of fat and fibrosis in the livers of control mice maintained on a regular diet (Figure 3A) in comparison with the livers of mice prenatally exposed to gutkha followed by the HFD for two weeks prior to sacrifice (Figure 3B). Sirius red staining (Figure 3C) of liver sections highlighted the observed ‘chicken-wire’ pattern of fibrosis typical of more advanced NAFLD or NASH. 

**Figure 4:**
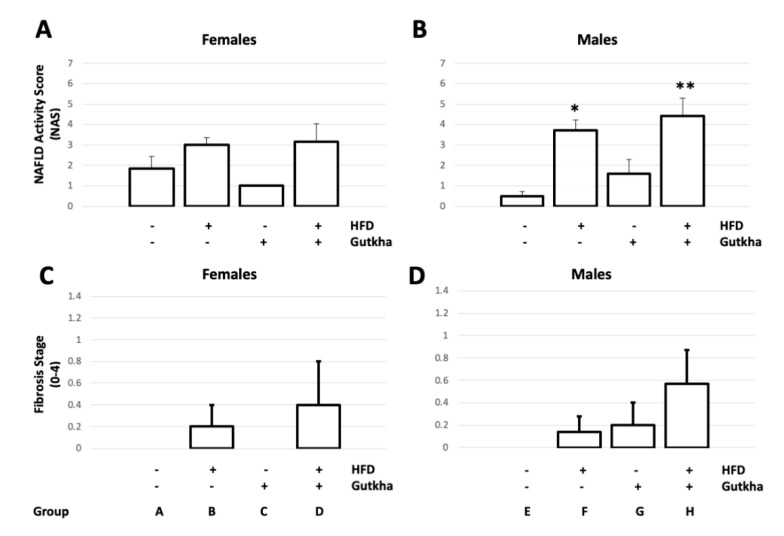
Pathologic Review of Liver Sections. (**A** & **B**) NAFLD activity scores (NAS) were significantly (*^,^** *p* < 0.05) higher in mice fed a HF diet (HFD). In male mice (**B**) gutkha exposure *in utero* further enhanced NAS. (**C** & **D**) Fibrosis stage was generally higher in response to a HFD, significantly so in male mice exposed *in utero* to gutkha and later fed a HFD. (Stage 1 represents low level fibrosis, Stage 0 = no fibrosis).

The mean NAS was significantly increased for the liver of male offspring fed the HFD (Groups G and H) as compared with those of mice maintained on a regular diet (Groups E and F) (Figure 4B). Of note, the observed mean NAS was the greatest for the male offspring (Group H) exposed to gutkha prenatally and fed the HFD for two weeks (Figure 4B). Additionally, the percentage of mice with a NAS > 4 (diagnostic of NASH) was also the highest (57%) in Group H. The mean NAS in female offspring followed a similar trend to that of the male NAS values, although the differences failed to reach statistical significance (Figure 4A).

As expected, given the short exposure period to the HFD, none of the mice developed hepatic fibrosis beyond stage 1 (Figure 4C,D), except one mouse in group H that had stage 2 fibrosis. As with mean NAS, the mean fibrosis score was the highest for male offspring (Group H) exposed to gutkha prenatally and subsequently fed the HFD for two weeks, but none of the relevant comparisons reached statistical significance. 

### 3.5. Blood Biochemistry 

ALT and AST serum levels are markers of hepatocellular injury and are sometimes mildly elevated in NAFLD. Serum lipid levels are generally increased by consumption of an HFD but may be decreased by liver disease. Since the normal range of these serum values differ by sex, comparisons were limited to groups of the same sex. The mean serum ALT levels were significantly increased in prenatally gutkha-exposed female offspring maintained on a control diet (Group B) versus non gutkha-exposed female offspring on a control diet (Group A), as shown in Table 2 (*p* < 0.05); these findings did not hold true for the male counterpart groups.

Both female and male offspring fed an HFD following prenatal gutkha exposure had significantly higher mean AST levels when compared to those fed an HFD alone (Groups C vs. D, *p* < 0.01 and Groups F vs. H, *p* < 0.01, respectively). The mean serum ALT, total cholesterol, and LDL levels were the highest in male offspring (Group H) prenatally exposed to gutkha followed by an HFD for two weeks prior to sacrifice; the same group also had the highest NAS and fibrosis stage. Interestingly, serum TRIG levels were significantly decreased in female and male offspring prenatally exposed to gutkha (regular diet) compared with non-gutkha controls (Groups A vs. B and Groups E vs. F, *p* < 0.05, respectively).

### 3.6. Pro-Fibrotic Cytokine (IL-6 and IL-1b) and Collagen (Col1A) mRNA Expression

**Figure 5:**
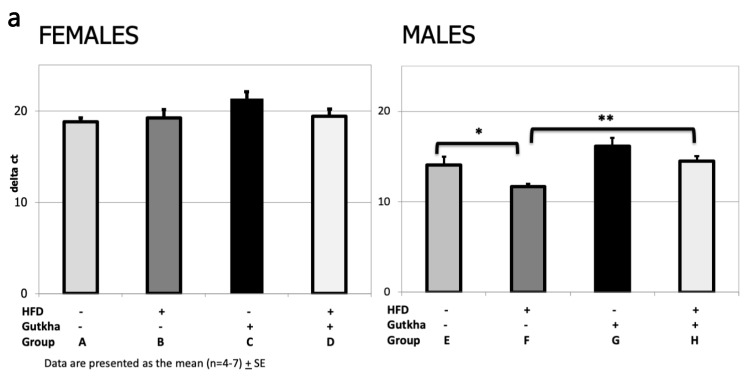

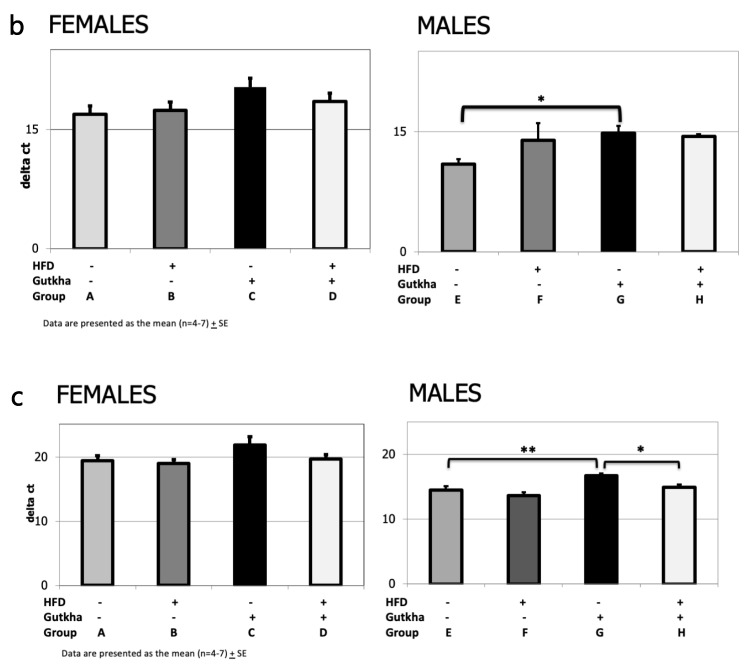
**a**–**c**. (**a**) Collagen 1 (Col1A1) mRNA gene expression is significantly (** *p* < 0.003) decreased in the livers of male offspring fed a HFD following prenatal gutkha exposure and significantly (* *p* < 0.03) increased in males fed a HFD alone. (**b**) Prenatal gutkha exposure alone significantly (* *p* < 0.01) decreases mRNA gene expression of IL-6 in the liver of male, but not female, offspring. (**c**) Prenatal gutkha exposure alone significantly (** *p* < 0.03) decreases mRNA gene expression of IL-1β in the liver of male offspring compared to sex- and diet-matched control counterparts; while male offspring prenatally exposed to gutkha followed by HFD have significantly (* *p* < 0.02) higher expression of IL-1β compared to sex- and exposure-matched control counterparts.

While Col1A mRNA expression was higher in male offspring fed the HFD compared to those continuously on the control diet, gutkha exposure appeared to suppress hepatic Col1A mRNA levels (Figure 5a). Male offspring (Group F) fed the HFD (without gutkha exposure) demonstrated a significant increase in mean Col1A1 mRNA expression when compared with male offspring without gutkha exposure (Group E) and fed a control diet and compared to male offspring (Group H) fed the HFD with prenatal gutkha exposure (Figure 5a). Group H mean Col1A levels were higher than Group G levels but failed to reach statistical significance. Gutkha exposure significantly decreased both IL-6 and IL-1b expression (Figure 5b,c, respectively) in male offspring on the control diet (Group G vs. E, *p* < 0.02) but not in male offspring subsequently fed the HFD (Group H vs. F, *p* > 0.05). In female offspring, no significant differences were observed in Col1A1, IL-6, and IL-1b mRNA levels between any of the groups. 

## 4. Discussion

Prenatal gutkha exposure exacerbated NAFLD in this study of first-generation adult murine offspring, which was influenced by both diet and sex; possibly, gutkha exposure also influenced sex outcomes. A high-fat diet was utilized to induce NAFLD changes in the adult offspring. The mechanism by which gutkha exacerbated NAFLD in the adult male offspring remains to be elucidated. Interestingly, hepatic cytokine levels were significantly affected by prenatal gutkha exposure in the adult male offspring on the control diet. An altered hepatic cytokine milieu could affect both inflammatory and fibrotic responses to the introduction of an HFD. The only ingredient/metabolite of gutkha measured in the serum of dams was cotinine, so the exact ingredient/metabolite(s) of gutkha which may have caused the altered hepatic cytokine milieu requires further research. The mean cotinine levels in the dam sera and amniotic fluid were similar to that found in the serum of active human smokers and are associated with dose-dependent epigenetic changes in DNA methylation [27,28]. This study provides evidence that nicotine (or its metabolite cotinine) crosses the placenta and could possibly play a role in the aforementioned hepatic changes. While these findings are consistent with prior reports indicating limited hepatic uptake of cotinine, as opposed to nicotine [29], they do demonstrate the placental passage of gutkha-associated nicotine products into the fetus. However, components of gutkha other than nicotine (e.g., arecoline and heavy metals such as lead, arsenic, copper, zinc [30] could also cross the placenta, resulting in fetal hepatotoxicity. Further studies are needed to determine which gutkha ingredient(s) could affect liver disease in adult offspring of mothers who use gutkha during pregnancy.

While maternal gutkha exposure during pregnancy did not significantly affect gestational parameters including maternal weight gain or birth weight, prenatal exposure to ST significantly reduced postnatal weight gain in both sexes from PND 9 through PND 12. While weight measurements were only performed up to PND 12, the absolute body weight was also reduced in female offspring prenatally exposed to gutkha at the time of sacrifice (14 weeks of age), suggesting an effect of prenatal gutkha exposure, particularly in females. While human studies have demonstrated a dose-dependent relationship between ST use during pregnancy and low birth weight [31], the gutkha-induced decrease in postnatal weight gain observed in this study is not as well established [15,32]. However, Quelhas et al. [33]) recently published a meta-analysisof growth outcomes in children (under 5 years of age), concluding that tobacco use by women during pregnancy has a negative impact on all growth outcomes measured (i.e., small for gestational age (SGA), length, and head circumference). The data presented here, along with those in Quelhas review, suggest an underlying need for additional research into factors affecting postnatal weight gain following prenatal tobacco use.

The significant increase in weight gain over the 2-week HFD exposure period suggests consumption of the HF chow by the mice. Human studies suggest that multiple ‘hits’ and/or ‘stressors’ are required to cause NASH and that epigenetics may play a significant role [34,35]. Our earlier study by Allina et al. [3]) supports the two-hit hypothesis by demonstrating that prenatal exposure to cigarette smoke followed by immune sensitization increased adult offspring susceptibility to liver disease. The current study also demonstrates that the effects of prenatal exposure to gutkha on adult offspring hepatic parameters are sex-dependent. Male offspring exposed prenatally to gutkha followed by an HFD beginning at 12 weeks of age, proved more susceptible than age- and diet-matched female offspring to gutkha-induced hepatic effects. Although data demonstrating sex-dependency following prenatal gutkha exposure are limited, investigations by Chen et al. [36] showed male mice to be more susceptible to hepatic changes following exposure to polyethylene-coated gold nanoparticles. In other cases, female mice have proven more susceptible to hepatic changes [37]. The metabolism of many toxicants is mediated by cytochrome P450 expression, which displays sex-dependent gene regulatory patterns in mice and humans [38,39]. Indeed, Fuscoe et al. [40] demonstrated that hepatic transcript profiles of drug-metabolizing enzymes, specifically, cytochrome P450s and transporters, can be used to predict sex-associated differences in drug metabolism. Filis et al. [41] also demonstrated sex-dependent alterations in liver protein expression in children of both sexes following maternal smoking during pregnancy. More detailed studies are needed to fully explore the sex differences in hepatic gene expression observed in adult offspring specifically following prenatal gutkha exposure. However, the increased NAS and serum AST levels are indicative of hepatic inflammation, which is shown in these studies to be associated with prenatal gutkha exposure and a subsequent HFD. 

Maternal cigarette smoking during pregnancy has been described as an important risk factor for many disease states in the offspring later in life, including fatty liver, hypertension, obesity, and type 2 diabetes [42,43,44]. These disorders are all facets of the metabolic syndrome, suggesting that gutkha use during pregnancy could have adverse impacts on related disease states. Former and current heavy smoking is also associated with NAFLD, and a synergistic effect is seen in active smokers in combination with an elevated body mass index (BMI). Furthermore, active and former cigarette smokers are not the only groups at risk for NAFLD. Women who never smoked but were ‘passive smokers’, exposed to smoke during both childhood and adulthood, had a 25% higher risk for NAFLD [45], suggesting an alternate pathway to exposure that may still yield serious outcomes later in life. 

Nicotine from maternal cigarette smoking is known to constrict intrauterine vessels [46] and cause fetal hypoxia [47]. Hypoxia, in turn, is known to alter fetal hepatic mRNA expression levels [48,49]. In the current study, cotinine was measured in maternal sera, amniotic fluid, and fetal liver tissue. Serum cotinine levels in dams were similar to those observed in human smokers [50,51]. As cotinine alters hepatocyte redox state ([52], which affects hepatocyte viability and gene expression [53], it is possible that fetal exposure to cotinine (or another nicotine metabolite) acts directly to affect hepatocytes and induce the same hepatic injury observed in the adult offspring of our study. It could be important to explore this pathway in future studies involving prenatal gutkha exposure. 

### Limitations

A limitation of this study was the relatively short HFD exposure period (2 weeks), which could have precluded the development of statistically significant differences in some cases. There was also some variability in the average weight gain of the offspring prior to sacrifice, particularly for the males, who, on average, had an overall negative weight gain from 12 to 14 weeks of age. The offspring in this study were an F2 generation and were fasted for 18 h prior to sacrifice, which potentially accounted for the variability observed. 

Furthermore, alternative strains of mice may be better suited for the analysis of steatohepatitis (e.g., C57BL/6). For example, the release of transaminases, critical for the identification and risk of NASH, differs between mouse strains in the following order: A/J > C57BL/6 > C3H/HeN = Balb/c = DBA/2 J [54]. The B_6_C_3_F_1_ mouse (CH3 × C57BL/6) was selected for these studies, given their routine use in reproductive/developmental toxicology studies. Building on the data presented here, future studies could incorporate an additional strain of mice (e.g., A/J, C57BL/6, C3H/HeN, or Balb/c) better suited to evaluate steatohepatitis susceptibility following prenatal ST exposure.

Our analysis demonstrates that changes in hepatic gene expression occur in response to prenatal gutkha exposure at a single timepoint. More extensive gene arrays and epigenetic analysis at multiple time points are needed to conclude a cause-and-effect relationship between prenatal gutkha exposure and fatty liver disease in adulthood. 

Extrapolating these results to other forms of ST or even other lots of gutkha is problematic, since a variety of ingredients (other than tobacco), including those not listed on the package, are found in these products. Nicotine and its metabolite cotinine are just the best-studied ingredients to date.

## 5. Conclusions

This study underscores the effects of an internationally relevant and commonly used ST product during pregnancy on postnatal offspring growth and hepatic risk factors later in life (adult). These data add to the increasing evidence that prenatal or early-life exposures can impact adult diseases. The results of these studies add substantially to the limited knowledge of the developmental origins of health and disease and of the toxicological effects of ST use during pregnancy. Data from such early-life studies can lead to potential intervention strategies and better health outcomes for adults, particularly those in regions where there is a strong cultural tie to ST products and/or a misperceived benefit to its continued use. Additional education, outreach, and cessation programming regarding tobacco use, specifically ST, which is often considered a ‘safer’ alternative to smoking, can make a huge difference in protecting vulnerable populations worldwide. 

## Figures and Tables

**Table 1:** Prenatal gutkha exposure followed by a high fat diet (HFD) significantly increased weight gain in male offspring.

Group	Sex	Gutkha	Diet	Average Absolute Wt Gain ^a^
				(grams)
**A**	**F**	**No**	**CTRL**	0.80* ± 0.52
**B**	**F**	**Yes**	**CTRL**	0.33 ± 0.44
**C**	**F**	**No**	**HF**	2.83* ± 0.47
**D**	**F**	**Yes**	**HF**	1.75 ± 0.88
**E**	**M**	**No**	**CTRL**	−0.39 ± 0.69
**F**	**M**	**Yes**	**CTRL**	0.70* ± 0.52
**G**	**M**	**No**	**HF**	0.82* ± 0.67
**H**	**M**	**Yes**	**HF**	3.08 ± 0.70

^a^ weight change over 2-week period (from 12–14 weeks of age) following introduction of HFD. Mean (n = 5–9 mice/treatment group) ± SE). * Significant differences: Group A vs. Group C (*p* < 0.03); Group F vs. Group H (*p* < 0.03) Group C vs. Group G (*p* < 0.04); Group G vs. Group H (*p* < 0.03).

**Table 2:** Serum cholesterol, ALT and AST levels were higher in mice fed a HFD. Gutkha exposure in utero significantly affected serum lipid, ALT and AST levels.

Group	Sex	Gutkha	Diet	ALT	AST	T. Chol	TRIG	HDL	LDL	HDL/LDL Ratio
A	F	No	CTRL	16.6* ± 0.7	45.3 ± 5.6	115.7 ± 13.5	23.0*± 6.8	84.5*± 10.0	14.7 ± 0.9	5.8 ± 0.6
B	F	Yes	CTRL	23.5* ± 2.6	52.1 ± 3.5	82.7 ± 9.9	8.5*± 1.8	54.5*± 4.9	12.4 ± 1.0	4.9 ± 0.5
C	F	No	HF	24.0 ± 1.3	47.6* ± 3.8	109.1 ± 16.0	20.4 ± 3.4	64.8 ± 5.9	18.7 ± 1.6	4.3 ± 0.3
D	F	Yes	HF	25.4 ± 2.3	62.5* ± 5.6	106.1 ± 3.1	19.9 ± 3.1	74.3 ± 3.4	18.7 ± 1.7	4.4 ± 0.4
E	M	No	CTRL	21.7 ± 2.5	32.2 ± 3.4	142.2 ± 9.8	45.5* ± 7.1	102.1 ± 7.6	16.1 ± 0.7	6.3 ± 0.4
F	M	Yes	CTRL	20.7 ± 1.8	28.0* ± 2.9	150.7 ± 14.8	23.4* ± 3.1	112.2 ± 8.8	16.7 ± 2.8	7.0* ± 0.5
G	M	No	HF	27.9 ±1.9	41.9 ± 3.3	175.1 ± 10.7	60.3 ± 8.0	124.6 ± 7.1	20.8 ± 1.1	6.1 ± 0.41
H	M	Yes	HF	28.3 ±3.0	43.3* ± 2.0	191.0 ± 8.3	48.4 ± 5.7	136.5 ± 6.1	25.0 ± 0.9	5.5* ± 0.3

Data presented as the mean (n = 5–9 mice per treatment groups) ± SE * Significant Differences for Each Sex:**ALT**: Group A vs. Group B (*p* < 0.05) **TRIG:** Group A vs. Group B (*p* < 0.05) Group E vs. Group F (*p* < 0.05) **AST:** Group C vs. Group D (*p* < 0.01) Group F vs. Group H (*p* < 0.01) **HDL:** Group A vs. Group B (*p* < 0.01) **HDL/LDL Ratio:** Group F vs. Group H (*p* < 0.03).

## Supplementary Materials

The following are available online at https://www.mdpi.com/1660-4601/17/21/7895/s1, Table S1: Supplemental Table of Primers.
