# Supplementary material for: Prenatal Exposure to Gutkha, a Globally Relevant Smokeless Tobacco Product, Induces Hepatic Changes in Adult Mice
Source: Int J Environ Res Public Health. 2020 Oct 28;17(21):7895. doi: 10.3390/ijerph17217895 (PMC7662769; doi:10.3390/ijerph17217895)
Supplement: Supplementary file 1 [file ijerph-17-07895-s001.pdf]

Supplemental Table of Primers

Gene	Strand	Sequence (5' --> 3')	Reference
Collagen 1A	Forward	CTGCTGGCAAAGATGGAGA	
	Reverse	ACCAGGAAGACCCTGGAATC	
IL-1β	Forward	GACGGCACACCCACCCT	
	Reverse	AAACCGTTTTTCCATCTTCTTCTT	
IL-6	Forward	CCAGAAACCGCTATGAAGTTCCT	
	Reverse	CACCAGCATCAGTCCCAAGA	
TNFα	Forward	TGTCTCAGCCTCTTCTCATTCC	
	Reverse	TGAGGGTCTGGGCCATAGAAC	
18S rRNA	Forward	CGGCTACCACATCCAAGGAA	Blum et al., 2009; 2015
	Reverse	CCTGTATTGTTATTTTTCGTCACTACCT	
